# Characterization of PAN-1, a Carbapenem-Hydrolyzing Class B β-Lactamase From the Environmental Gram-Negative *Pseudobacteriovorax antillogorgiicola*

**DOI:** 10.3389/fmicb.2019.01673

**Published:** 2019-07-23

**Authors:** Nicolas Kieffer, Laurent Poirel, Claudine Fournier, Brad Haltli, Russel Kerr, Patrice Nordmann

**Affiliations:** ^1^ Medical and Molecular Microbiology Unit, Department of Medicine, Faculty of Science, University of Fribourg, Fribourg, Switzerland; ^2^ Department of Medicine, INSERM European Unit (IAME, France), University of Fribourg, Fribourg, Switzerland; ^3^ Department of Medicine, Swiss National Reference Center for Emerging Antibiotic Resistance (NARA), University of Fribourg, Fribourg, Switzerland; ^4^ Department of Biomedical Sciences, Atlantic Veterinary College, Charlottetown, PE, Canada; ^5^ Department of Medicine, Institute for Microbiology, University of Lausanne and University Hospital Centre, Lausanne, Switzerland

**Keywords:** carbapenemase, metallo-β-lactamase, PAN-1, environment, *Pseudobacteriovorax antillogorgiicola*

## Abstract

The gene encoding the metallo-β-lactamase (MβL) PAN-1 was identified in the genome of the environmental Gram-negative species *Pseudobacteriovorax antillogorgiicola*. PAN-1 shares 57% amino-acid identity with the acquired MβL SPM-1, its closest relative. Kinetic parameters performed on purified PAN-1 showed it displayed a hydrolytic activity toward most β-lactams including carbapenems but spared cefepime and aztreonam. These results further highlight that environmental bacterial species may be reservoirs of MβL encoding genes.

## Introduction

Class B carbapenemases are metallo-β-lactamases (MβLs) that belong to a highly diverse family of enzymes. They are characterized by their hydrolytic activity against β-lactams including carbapenems. The MβLs can be differentiated into three subclasses, B1, B2, and B3, based on the nature of the amino acid residues involved in binding the zinc ion required in the catalytic site of these enzymes (Meini et al., 2014). The B1 subclass comprises the most clinically significant and acquired MβLs such as NDM-1, VIM-1, IMP-1, and SPM-1 (Meini et al., 2014). When acquired, the corresponding genes are usually associated to mobile genetic elements such as class 1 integrons and composite transposons, which are themselves often located onto plasmids. These genetic structures can explain their frequent diffusion among clinical species such as *Enterobacteriaceae*, *Pseudomonas* spp., and *Acinetobacter* spp.

The origin of all clinically relevant MβLs remains unknown. *In silico* tools can be useful in order to identify putative clinical relevant MβL progenitors within DNA sequence databases. In this study, we characterized a B1 MβL showing relatedness with the SPM-1 enzyme in which gene was identified in the genome of an environmental species named *Pseudobacteriovorax antillogorgiicola*.

## Materials and Methods

### *In silico* Sequence Analyses

The *bla*_PAN-1_ was identified using the NCBI blast alignment tool excluding all clinical relevant species such as *Enterobacteriaceae*, *Acinetobacter* spp., and *Pseudomonas* spp. Phylogenetic analysis was performed using the alignment software SeaView (Prabi, La Doua, France).

### Bacterial Isolates and Susceptibility Testing

Strain *P. antillogorgiicola* RKEM611^T^ was isolated on marine agar (Carlroth AG, Arlesheim, Switzerland) at 30°C for 48 h. Antimicrobial susceptibility testing was performed according to the standard disk diffusion method on marine agar for the RKEM611^T^ isolate and on Muller-Hinton (MH) agar (Bio-Rad, Cressier, Switzerland) for the *Escherichia coli* clones, respectively. The interpretation of the susceptibility profile was determined following the CLSI recommendations (Clinical and Laboratory Standards Institute, 2018). Minimal inhibition concentrations (MICs) were determined by broth microdilution using cation-adjusted MH broth.

### Cloning of the *bla*_PAN-1_ Gene

The *bla*_PAN-1_ gene was cloned into the pTOPO-kan^R^ cloning vector using the pCR-Blunt TOPO cloning kit (Invitrogen, Illkirch, France) using specific primers (PAN-1-Fw: 5′-CAAGGTAACGGAGAAAATTG-3′ and PAN-1-Rv: 5′-GGCTAACCTAGAATTTAGTC-3′) including the full gene in order to express the whole protein. The resulting recombinant plasmid was electroporated into the *Escherichia coli* TOP10 (pPAN-1-TOP) and the OmpC-OmpF porin deficient *E. coli* HB4 (pPAN-1-HB4; to mirror a strain with permeability defects) strains, respectively.

### Purification of the PAN-1 Protein

Purification of the PAN-1 β-lactamase was carried out by ion-exchange chromatography. *E. coli* TOP10 (pPAN-1) was grown in 4 L of Luria-Bertani (LB) broth containing 100 μg/ml of ampicillin and 25 μg/ml of kanamycin. The overnight culture was centrifuged, and the obtained pellet was resuspended in piperazine buffer pH 11.2 (0.1 M) and sonicated using a Vibra cell™ 75,186 sonicator (Thermo Fisher Scientific). After filtration using a 0.22-μm nitrocellulose filter, the crude extract was loaded in a Q-Sepharose column connected to an ÄKTAPrime chromatography system (GE Healthcare) and eluted with a linear pH gradient using piperazine buffer at pH 6.5 (0.1 M). The presence of β-lactamase in the different elution fractions was monitored using nitrocefin (200 μM). All positive fractions were pooled and dialyzed overnight at 4°C against HEPES buffer (0.1 M, pH 7.5) supplemented with ZnSO_4_ (50 mM). The protein concentrations were measured using Bradford reagent (Sigma-Aldrich, Buchs, Switzerland).

### Enzymatic Characterization of PAN-1

Kinetic measurements were performed at room temperature in HEPES buffer supplemented with zinc using a UV visible Ultrospec 2100 pro spectrophotometer (Amersham Biosciences, Buckinghamshire, UK).

## Results and Discussion

**Figure 1:**
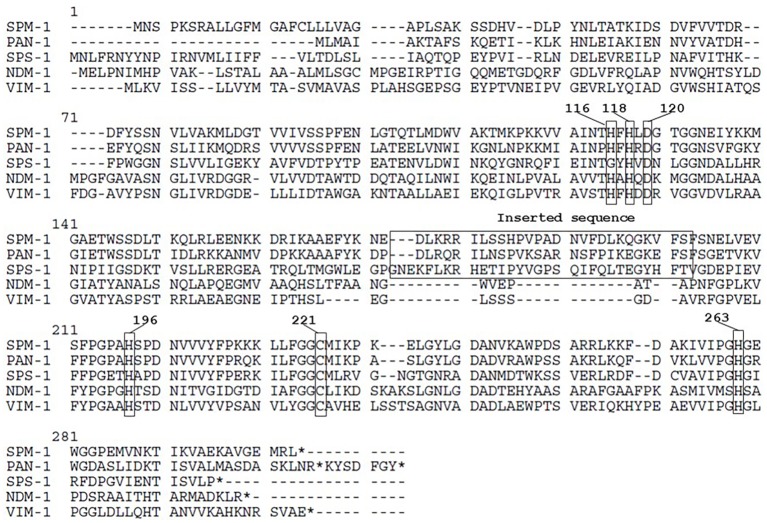
Amino acid sequence comparison of PAN61 and other metallo-β-lactamases. Conserved amino acid residues corresponding to the binding site with the zinc ion required for the hydrolytic activity and the inserted sequence feature are bracketed. Numbers in bold represent the BBL numeration. * means stop codon.

**Figure 2:**
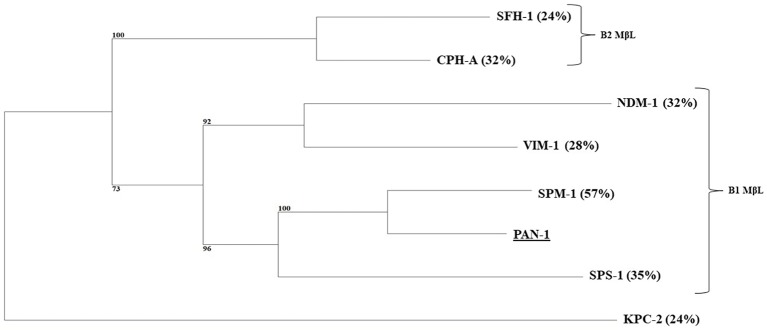
Phylogenetic tree obtained for clinical relevant MβLs and PAN-1 by distance method using neighbor-joining algorithm (SeaView version 4 software). Branch lengths are drawn to scale and are proportional to the number of amino acid substitutions with 500 bootstrap replications. The distance along the vertical axis has no significance. Percentage of amino acid identity shared with the PAN-1 enzyme is indicated in brackets. The tree is rooted with the KPC-2 enzyme.

*P. antillogorgiicola* is an environmental species recovered in 2011 from a soft coral named *Antillogorgia elisabethae* in the Bahamas sea (McCauley et al., 2015). This isolate is so far the only representative of this species and belong to the *Bdellovibrionales* order. Species belonging to this order are known to exhibit a predatory feature meaning that they are able to infect other Gram-negative rod in their environment. Analysis of the *P. antillogorgiicola* RKEM611^T^ genome sequence (accession number: FWZT01000001.1) revealed a putative metallo-β-lactamase (MβL) gene, named *bla*_PAN-1_, located on the chromosome of that strain. Further analysis of the genetic environment did not identify any mobile element suggesting that this gene is part of the core genome of that species. The GC content of the gene was 42%, which was similar to the whole genome of that species. The *bla*_PAN-1_ gene encodes a putative enzyme (PAN-1) of 260 amino acids, sharing 57% amino acid identity with the acquired SPM-1 MβL known to be widespread in *Pseudomonas aeruginosa* in Brazil (Labarca et al., 2016). Despite some significant sequence heterogeneity with SPM-1, the protein alignment between the two enzymes revealed conserved amino acid residues known to be crucial for the hydrolytic activity of MβLs, such as the histidine residues 116, 118, 196, and 263, the aspartic acid residue 120, and the cysteine residue 221, respectively (Figure 1; Meini et al., 2014). Phylogenetic analysis using the alignment software SeaView showed that PAN-1 is the closest relative enzyme to SPM-1 (Figure 2) and to the recently reported SPS-1 B1 MβL (Cheng et al., 2018). Interestingly, PAN-1 and SPS-1 exhibited a central inserted sequence that was considered as a unique feature of the SPM-1 MβL (Figure 1; Murphy et al., 2006; Meini et al., 2014; González et al., 2018). Antimicrobial susceptibility testing showed that isolate RKEM611^T^ was susceptible to most β-lactams including penicillins, carbapenems, and broad-spectrum cephalosporins such as cefotaxime, cefoxitin, and cephalothin but was resistant to ceftazidime, cefepime, temocillin, and aztreonam. Nonetheless, no enzymatic activity toward these latter β-lactams was detected by UV spectrophotometry using a crude enzymatic extract from this environmental isolate (data not shown). This result may be related to a weak expression of β-lactamase PAN-1 in the isolate.

Minimal inhibitory concentrations (MICs) were determined for both *E. coli* TOP10 (pPAN-1) and *E. coli* HB4 (pPAN-1) using broth microdilution method. According to the CLSI guidelines (Clinical and Laboratory Standards Institute, 2018), *E. coli* TOP10 (pPAN-1) showed resistance to amino- and carboxypenicillins and their derivatives containing a β-lactamase inhibitor; resistance to cephalosporins such as cephalothin, cefoxitin, cefotaxime, and ceftazidime; and intermediate resistance to carbapenems and piperacillin but remained susceptible to cefepime and aztreonam. This resistance profile was fully consistent with the production of a MβL. Noteworthy, the expression of the *bla*_PAN-1_ gene in *E. coli* HB4 conferred clinical resistance toward meropenem and ertapenem (Table 1; Clinical and Laboratory Standards Institute, 2018). This latter strain was actually tested in order to mirror the expression of PAN-1 in a clinical strain exhibiting decreased permeability. Indeed, the association between acquired β-lactamase and permeability defects is commonly observed in clinical isolates.

**Table 1:** MICs of β-lactams.

MICs (μg/ml)					
Antibiotics	*E. coli* TOP10 (pPAN-1)	*E. coli* HB4 (pPAN-1)	*E. coli* TOP10	*E. coli* HB4^*^	*E. coli* TOP10 (pSPM-1)
Amoxicillin	**128**	>**128**	2	16	>**128**
Amoxicillin + clavulanic acid^a^	**128**	**>128**	1	16	**>128**
Piperacillin	32	64	1	8	64
Cephalothin	**128**	>**128**	4	**128**	>**128**
Cefoxitin	**128**	>**128**	2	64	>**128**
Ceftazidime	**32**	**64**	0.125	1	>**128**
Cefotaxime	**4**	**8**	0.06	0.5	**64**
Cefepime	0.5	2	0.125	0.5	**16**
Aztreonam	0.06	0.38	0.06	0.38	0.06
Imipenem	0.5	0.5	0.06	0.06	0.5
Meropenem	0.25	**4**	0.06	0.25	0.5
Ertapenem	0.5	**2**	0.06	1	**4**

*Light gray shaded MICs represent values of intermediate profile susceptibility, and boldened MIC values correspond to resistance according to the CLSI guidelines*.

a *Clavulanic acid was added at a fixed concentration of 2 μg/ml*.

* *E. coli HB4 is a porin-deficient strain*.

Kinetic parameters determination confirmed the MβL property of PAN-1. Results obtained showed that this enzyme was able to hydrolyze numerous β-lactams. For cephalosporins, the lowest *K*_m_ values were observed for cefoxitin and cephalothin (13.3 and 32.2 μM, respectively), while the highest *K*_m_ value was found to be cefotaxime with a *K*_m_ of 245.7 μM. Kinetic measurements showed that PAN-1 had no affinity against aztreonam and cefepime. The highest catalytic efficiencies (*k*_cat_/*K*_m_) were found for cephalothin and cefoxitin, whereas the lowest were observed with ceftazidime and cefotaxime, respectively. Among carbapenems, PAN-1 showed a better catalytic efficiency against imipenem that was approximately 1.6- and 11.9-fold higher than those against meropenem and ertapenem, respectively. Altogether, kinetic results obtained for that Ambler class B carbapenemase are in agreement with the results of the antimicrobial susceptibility testing. Nonetheless, the lack of detection of hydrolytic activity toward cefepime may be considered as odd since PAN-1 confers a slight increase of MIC in *E. coli* clones (Table 1).

Our study characterized a novel MβL from an environmental isolate recovered from a coral. This enzyme showed the highest amino acid identity with the class B carbapenemase SPM-1 being its closest relative after the recently reported SPS-1 enzyme (Figure 2; Cheng et al., 2018)(. Interestingly, the amino acid sequence of PAN-1 exhibits an inserted sequence that was also described in SPM-1 (Murphy et al., 2003), being an original feature comparing to other subclass B1 MβLs (Figure 1). Several studies hypothesized that this inserted sequence might be involved in the restriction of substrates spectrum of subclass B2 MβLs, suggesting that SPM-1, SPS-1, and PAN-1 may have evolved from a common ancestor compared to subclass B2 MβLs (Cheng et al., 2018; González et al., 2018).

The β-lactamase PAN-1, like SPM-1 and other MβLs, is capable of hydrolyzing penicillins, cephalosporins, and carbapenems, sparing monobactams. However, unlike SPM-1, PAN-1 was not able to confer resistance toward cefepime or ertapenem once expressed in a susceptible *E. coli* isolate (Table 1). Comparison of the kinetic parameters of the SPM-1 enzyme confirmed these dissimilarities (Murphy et al., 2003). Unlike PAN-1, SPM-1 was indeed described to have higher catalytic efficiencies against cefepime and most β-lactams in general, except against aztreonam. The expression of the *bla*_PAN-1_ gene in a porin-deficient strain led to very high MIC values for β-lactams, highlighting the potential impact of this gene if expressed in such background.

**Table 2:** Kinetic parameters of the PAN-1 metallo-β-lactamase.

Antibiotics	*k*_cat_ (s^−1^)	*K*_m_ (μM)	*k*_cat_/*K*_m_ (mM^−1^ s^−1^)
Penicillin G	23.6	161	146
Piperacillin	73.6	1074.2	68
Cephalothin	6.9	32.2	214
Cefoxitin	1.9	13.3	143
Ceftazidime	8.1	144.4	56
Cefotaxime	3.9	245.7	16
Cefepime	<0.01	>1,000	ND
Aztreonam	<0.01	>1,000	ND
Imipenem	43.8	470.5	93.1
Meropenem	22.9	386	59
Ertapenem	2.9	368.5	7.8

The high susceptibility of the environmental *P. bacteriovorax* isolate to β-lactams suggests that the *bla*_PAN-1_ gene is poorly or not expressed in its original host. Previous studies about progenitors of class D carbapenemases showed a similar phenomenon. For instance, the progenitors of the OXA-23 and OXA-181 β-lactamases (namely *Acinetobacter radioresistens* and *Shewanella xiamenensis*, respectively) showed high susceptibility to β-lactams (Poirel et al., 2008; Potron et al., 2011). These results may also be explained by the single copy number of those β-lactamase genes in their original host and raise questions about the function of those β-lactamases in their progenitor species.

## Data Availability

The datasets generated for this study can be found in GENBANK, FWZT01000001.1.

## Author Contributions

PN, LP, and NK designed the experiments. BH and RK provided the bacterial isolate. NK and CF performed the experiments and analyzed the data. PN, LP, and NK wrote the manuscript.

### Conflict of Interest Statement

The authors declare that the research was conducted in the absence of any commercial or financial relationships that could be construed as a potential conflict of interest.
